# The complete mitochondrial genome of *Eremias yarkandensis* (Reptilia, Squamata, Lacertidae) from Kyrgyzstan

**DOI:** 10.1080/23802359.2022.2047119

**Published:** 2022-03-02

**Authors:** Song Wang, Jinlong Liu, Marina A. Chirikova, Bin Zhang, Xianguang Guo

**Affiliations:** a Chengdu Institute of Biology, Chinese Academy of Sciences, Chengdu, China; b College of Life Sciences & Technology, Inner Mongolia Normal University, Hohhot, China; c Institute of Zoology of Republic of Kazakhstan, Almaty, Kazakhstan

**Keywords:** Kyrgyzstan, mitochondrial genome, next-generation sequencing, phylogenetic tree, *Eremias*, viviparity

## Abstract

The Yarkand racerunner, *Eremias yarkandensis* Blandford, 1875, is only distributed in China and Kyrgyzstan. Its complete mitogenome was determined by next-generation sequencing for the first time. The mitogenome was 18,743 bp in length, including 13 protein-coding genes (PCGs), two ribosomal RNA genes, 22 transfer RNA genes, and 1 control region. Its gene arrangement was similar to the typical mtDNA of vertebrates. The 13 concatenated PCGs were used to perform Bayesian phylogenetic analyses together with several congeners as well as two representative species of *Lacerta* with mitogenome data in GenBank. The resulting phylogenetic tree recovered the monophyly of both *Eremias* and its viviparous group, with *E. yarkandensis* being more closely related to *E. przewalskii* than to *E. dzungarica*. The mitogenome of *E. yarkandensis* will provide fundamental data for the exploration of the mitogenome evolution in racerunners.

The viviparous Yarkand racerunner, *Eremias yarkandensis* Blandford, 1875, is endemic to East Alai of Kyrgyzstan and Xinjiang Uyghur Autonomous Region of China, with terra typica in the Yarkand County, Kashgaria, China. Historically, it was assigned to subspecies status of *Eremias multiocellata yarkandensis* (Boulenger 1921; Schmidt 1926; Szczerbak 1974; Eremchenko et al. 1992). However, its status was confirmed as distinct biological species through long-term research including hybridization experiments (Eremchenko and Panfilov 1999). To date, little is known about its genetic affinities with other viviparous species in genus *Eremias* albeit with limited understanding of viviparity evolution in racerunner lizards (Guo et al. 2011; Orlova et al. 2017; Liu et al. 2021).

In this study, we determined for the first time the complete sequence of the mitogenome of *E. yarkandensis* by next-generation sequencing through the Illumina NovaSeq platform. A female adult (voucher number Guo4719) was collected from Kyrgyzstan (N39.64809°, E73.86512°; 2963 meters above sea level) in August 2014. The collection of lizard used for this study obeyed the Law ‘On the Animal World’ No. 59 of Kyrgyzstan, and followed the guidelines in the Institute of Biology and Soil, National Academy of Science of the Kyrgyz Republic. Its liver tissue was fixed with 95% ethanol, and stored at −20 °C in the Chengdu Institute of Biology (CIB), Chinese Academy of Sciences (Contact person: Xianguang Guo, E-mail: guoxg@cib.ac.cn). A small amount of liver tissue was shipped to Genepioneer Biotechnologies (Nanjing, China) for genomic extraction and 150-base-pair paired-end library construction as well as sequencing. The CIB Animal Care and Use Committee approved all experimental procedures (No. 20200767).

The raw data were processed with fastp v.0.20.0 (Chen et al. 2018), by trimming adapters and primers, filtering reads with phred quality < Q5, and filtering reads with N base number > 5. *De novo* assembly of clean data (6,996,357,900 pair-end reads) was performed using SPAdes v.3.10.1 (Bankevich et al. 2012). The average coverage of reads aligned on the mitogenome was 625.4147. We used a published sequence (GenBank accession number MW250881; Wang et al. 2021) as the reference for queries to assemble the mitogenome. Subsequently, the mitogenome was annotated with the MITOS WebServer (Bernt et al. 2013). Specifically, 22 tRNA genes were identified by using the web server of tRNA scan-SE (Lowe and Chan 2016). The base composition was calculated in MEGA v.7.0 (Kumar et al. 2016).

The gene content, arrangement, and composition exhibited a typical vertebrate mitogenome feature. All of 37 genes were completely recovered including two ribosomal RNA genes, 13 protein-coding genes (PCGs), 22 transfer RNA (tRNAs), and a control region (CR or D-loop). The mitogenome of *E. yarkandensis* was 18,743 bp in length, which was composed of 28.4%(T), 27.2%(C), 31.1%(A), 13.3%(G). In 13 PCGs, the shortest was ATP8 gene (162 bp) and the longest was ND5 (1824 bp). Eleven PCGs used ATG as start codons, the remaining two (COX1, ND1) used GTG as start codons. As for stop codons, six PCGs (ND1, ND4L, ND5, ATP6, ATP8, Cytb) used TAA; five PCGs (ND2, ND3, ND4, COX2, COX3) used T as an incomplete stop codon; two PCGs (COX1, ND6) used AGG. In addition, 12S rRNA, 16S rRNA, and D-loop were 951 bp, 1559 bp, and 3339 bp, respectively. The majority of the genes in the mtDNA of *E. yarkandensis* were distributed on H-strand, except for ND6 gene and eight tRNAs (tRNA-Glu, Ala, Asn, Cys, Tyr, Ser^[UGA]^, Gln, and Pro).

The Bayesian phylogenetic tree was used to assess mitochondrial sequence authenticity of *E. yarkandensis* and its phylogenetic placement by using the concatenated 13 PCGs of *E. yarkandensis* together with two other representative *Lacerta* lizards in GenBank. Gene partitioning, model selection, and tree reconstruction were made in the plug-in programs of PhyloSuite v.1.2.1 (Zhang et al. 2020). PartitionFinder v.2.1.1 was used to select the best-fitting substitution models and partitioning schemes with the Bayesian information criterion (Lanfear et al. 2017). Bayesian inference was conducted using MrBayes v.3.2.6 (Ronquist et al. 2012). Two independent runs were carried out with four Monte Carlo Markov chains (MCMCs) for 10 million generations with parameters and topologies sampled every 1000 generations. The convergence of the runs was assessed by the standard deviation of split frequencies (<0.01). A 50% majority-rule consensus tree and posterior probability (PP) of clades were assessed by combining the sampled trees from the two independent runs after a 25% burn-in phase.

As shown in Figure 1, the monophyly of both genus *Eremias* and its viviparous group was recovered with strong support (Guo et al. 2011; Orlova et al. 2017). *Eremias yarkandensis* was more closely related to *E. przewalskii* than to *E. dzungarica* albeit with moderate support (PP = 0.81). The mitogenome of *E. yarkandensis* will provide fundamental data for the exploration of the mitogenome evolution in racerunners.

**Figure 1.**
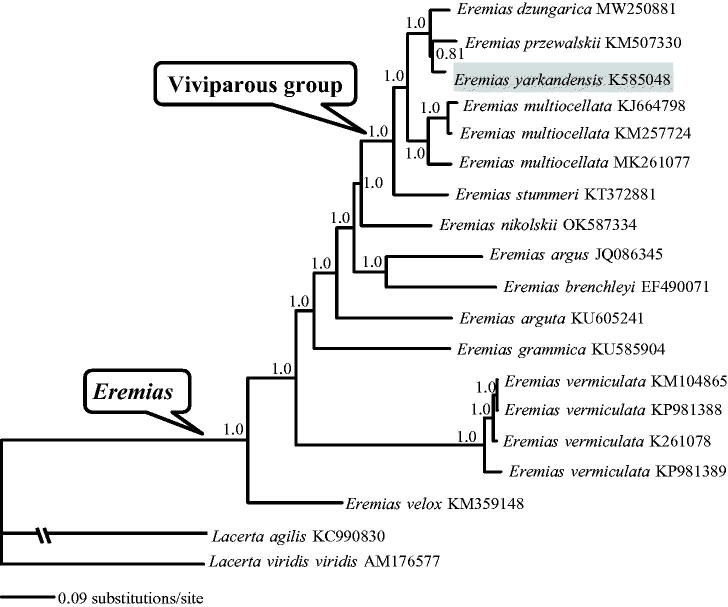
A majority-rule consensus tree inferred from Bayesian inference using MrBayes with the best model for each partition, based on the PCGs of *Eremias* spp. and two other representative *Lacerta* lizards retrieved from GenBank. The phylogenetic placement of *E. yarkandensis* is highlighted. GenBank accession numbers are given with species names. Node numbers show Bayesian posterior probabilities. Branch lengths represent means of the posterior distribution.

## Data Availability

The data that support the findings of this study are openly available in NCBI at https://www.ncbi.nlm.nih.gov/nuccore/OK585048, reference number OK585048 . The associated BioProject, SRA, and Bio-Sample numbers are PRJNA773190, SRR16508134, and SAMN22445465, respectively.

## References

[CIT0001] Bankevich A, Nurk S, Antipov D, Gurevich A, Dvorkin M, Kulikov AS, Lesin V, Nikolenko S, Pham S, Prjibelski A, et al. 2012. SPAdes: a new genome assembly algorithm and its applications to single-cell sequencing. J Comput Biol. 19(5):455–477. 22506599 10.1089/cmb.2012.0021 PMC3342519

[CIT0002] Bernt M, Donath A, Juhling F, Externbrink F, Florentz C, Fritzsch G, Pütz J, Middendorf M, Stadler PF. 2013. MITOS: improved *de novo* metazoan mitochondrial genome annotation. Mol Phylogenet Evol. 69(2):313–319. 22982435 10.1016/j.ympev.2012.08.023

[CIT0003] Boulenger GA. 1921. Monograph of the lacertidae. Vol. 2. London, UK: Trustees of the British Museum of Natural History.

[CIT0004] Chen S, Zhou Y, Chen Y, Gu J. 2018. fastp: an ultra-fast all-in-one fastq preprocessor. Bioinformatics. 34(17):i884–i890. 30423086 10.1093/bioinformatics/bty560 PMC6129281

[CIT0005] Eremchenko VK, Panfilov AM. 1999. Taxonomic situation of multiocellated racerunner of the “multiocellata”-complex of Kyrgyzstan and neighbor China (Sauria: Lacertidae: *Eremias*). Sci New Tech. 4:112–124. (in Russian with English abstract).

[CIT0006] Eremchenko VK, Panfilov AM, Tzarinenko EI. 1992. *Eremias multiocellata* complex: solution of some problems in systematics of the multiocellated racerunners of Kyrgyzstan (Sauria, Lacertidae, *Eremias*.). In: conspectus of the researches on cytogenetics and systematics of some Asiatic species of scincidae and lacertidae. Kiev: Ilim, Bishket (in Russian); p. 65–80.

[CIT0007] Guo X, Dai X, Chen D, Papenfuss TJ, Ananjeva NB, Melnikov DA, Wang Y. 2011. Phylogeny and divergence times of some racerunner lizards (Lacertidae: *Eremias*) inferred from mitochondrial 16S rRNA gene segments. Mol Phylogenet Evol. 61(2):400–412. 21767655 10.1016/j.ympev.2011.06.022

[CIT0008] Kumar S, Stecher G, Tamura K. 2016. MEGA7: molecular evolutionary genetics analysis version 7.0 for bigger datasets. Mol Biol Evol. 33 (7):1870–1874. 27004904 10.1093/molbev/msw054 PMC8210823

[CIT0009] Lanfear R, Frandsen PB, Wright AM, Senfeld T, Calcott B. 2017. PartitionFinder 2: new methods for selecting partitioned models of evolution for molecular and morphological phylogenetic analyses. Mol Biol Evol. 34(3):772–773. 28013191 10.1093/molbev/msw260

[CIT0010] Liu J, Dujsebayeva T, Chirikova M, Gong X, Li D, Guo X. 2021. Does the Dzungarian racerunner (Eremias dzungarica Orlova, Poyarkov, Chirikova, Nazarov, Munkhbaatar, Munkhbayar & Terbish, 2017) occur in China? Species delimitation and identification with DNA barcoding and morphometric analyses. Zool Res. 42(3):287–293. 33880891 10.24272/j.issn.2095-8137.2020.318 PMC8175952

[CIT0011] Lowe TM, Chan PP. 2016. tRNAscan-SE On-line: integrating search and context for analysis of transfer RNA genes. Nucleic Acids Res. 44 (W1):W54–W57. 27174935 10.1093/nar/gkw413 PMC4987944

[CIT0012] Orlova VF, Poyarkov NA, Chirikova MA, Nazarov RA, Munkhbaatar M, Munkhbayar K, Terbish K. 2017. MtDNA differentiation and taxonomy of central Asian racerunners of *Eremias multiocellata*-*E*remias *przewalskii* species complex (Squamata, Lacertidae). Zootaxa. 4282(1):1–42.

[CIT0013] Ronquist F, Teslenko M, Mark PVD, Ayres DL, Darling A, Hohna S, Larget B, Liu L, Suchard MA, Huelsenbeck JP. 2012. MrBayes 3.2: efficient Bayesian phylogenetic inference and model choice across a large model space. Syst Biol. 61(3):539–542. 22357727 10.1093/sysbio/sys029 PMC3329765

[CIT0014] Schmidt KP. 1926. Amphibians and reptiles of the James Simpson-Roosevelt Asiatic Expedition. Field Mus Nat Hist Zool. 12:167–173.

[CIT0015] Szczerbak NN. 1974. Yashchurki Palearktiki (The Palearctic Racerunners). Kiev: Naukova Dumka Press (in Russian).

[CIT0016] Wang S, Liu J, Zhang B, Guo X. 2021. The complete mitochondrial genome of *Eremias dzungarica* (Reptilia, Squamata, Lacertidae) from the Junggar Basin in Northwest China. Mitochondrial DNA B Resour. 6(7):2012–2014. 34189268 10.1080/23802359.2021.1923417 PMC8208112

[CIT0017] Zhang D, Gao F, Li WX, Jakovlic I, Zou H, Zhang J, Wang GT. 2020. PhyloSuite: an integrated and scalable desktop platform for streamlined molecular sequence data management and evolutionary phylogenetics studies. Mol Ecol Resour. 20(1):348–355. 31599058 10.1111/1755-0998.13096

